# Unraveling heme detoxification in the malaria parasite by *in situ* correlative X-ray fluorescence microscopy and soft X-ray tomography

**DOI:** 10.1038/s41598-017-06650-w

**Published:** 2017-08-08

**Authors:** Sergey Kapishnikov, Daniel Grolimund, Gerd Schneider, Eva Pereiro, James G. McNally, Jens Als-Nielsen, Leslie Leiserowitz

**Affiliations:** 1 Soft Matter and Functional Materials, Helmholtz-Zentrum Berlin, Albert-Einstein-Str. 15, 12489 Berlin, Germany; 2 Niels Bohr Institute, University of Copenhagen, Universitetsparken 5, 2100 Copenhagen, Denmark; 3 Paul Scherrer Institute, 5232 Villigen, PSI Switzerland; 4 Institute of Physics, Humboldt University, Newton str. 15, 12489 Berlin, Germany; 5 ALBA Synchrotron Light Source, MISTRAL Beamline–Experiments Division, 08290 Cerdanyola del Valles, Barcelona Spain; 6 Department of Materials and Interfaces, Weizmann Institute of Science, Rehovot, 76100 Israel

## Abstract

A key drug target for malaria has been the detoxification pathway of the iron-containing molecule heme, which is the toxic byproduct of hemoglobin digestion. The cornerstone of heme detoxification is its sequestration into hemozoin crystals, but how this occurs remains uncertain. We report new results of *in vivo* rate of heme crystallization in the malaria parasite, based on a new technique to measure element-specific concentrations at defined locations in cell ultrastructure. Specifically, a high resolution correlative combination of cryo soft X-ray tomography has been developed to obtain 3D parasite ultrastructure with cryo X-ray fluorescence microscopy to measure heme concentrations. Our results are consistent with a model for crystallization via the heme detoxification protein. Our measurements also demonstrate the presence of considerable amounts of non-crystalline heme in the digestive vacuole, which we show is most likely contained in hemoglobin. These results suggest a tight coupling between hemoglobin digestion and heme crystallization, highlighting a new link in the crystallization pathway for drug development.

## Introduction

Malaria is a severe disease caused by parasites of the genus *Plasmodium*. As part of its life cycle, the parasite consumes hemoglobin from red blood cells leading to the release of iron-containing heme molecules. Heme is toxic due to the reactivity of iron, so the parasite has developed mechanisms to detoxify it^1–6^. These detoxification mechanisms have been the target of drug development, since blocking heme detoxification will kill the parasite^3, 6–9^. There are at least three pathways for quinoline-family drugs and artemisinin proposed and discussed in the literature, namely interference with hemozoin nucleation by binding to the existing crystals^3, 9, 10^, by complexing with heme^6, 11, 12^, and interference with hemoglobin binding to falcipain 2 protease^6^.

The primary mechanism of heme detoxification is the formation of inert hemozoin crystals in the parasite’s digestive vacuole. However, there is considerable debate about how these hemozoin crystals form. The synthetic form of hemozoin can be obtained by a variety of methods under non-physiological conditions^13–15^. In the parasite, it has been suggested that heme crystallization is promoted by confining it to a lipid environment^9, 16–19^ or by catalyzing it with a protein^2, 6, 20^. Which, if any, of these mechanisms is actually used by the parasite remains uncertain, and this has hampered the development of new antimalarial drugs that could efficiently target heme detoxification. One way to identify the crystallization mechanism is to measure the rate of heme crystallization *in vivo*, since the different models proposed for heme crystallization predict vastly different rates.

A related issue critical for heme detoxification is how hemoglobin degradation and heme crystallization rates are coordinated within the parasite such that free heme levels remain negligible. Clearly, if hemoglobin degradation occurs faster than crystallization, free heme will accumulate in the digestive vacuole, and kill the parasite. Thus to avoid release of excess heme, non-crystalline heme must either be stored in the parasite or degradation of hemoglobin must be tightly coupled to heme crystallization. Distinguishing between these different options requires a method to measure the amounts of hemoglobin and non-crystalline heme in the digestive vacuole.

To address these key questions in heme detoxification, methods for a quantitative *in situ* biochemistry have been developed and applied to measure iron levels in the malaria parasite. Specifically, we have combined two forms of X-ray microscopy, each of which can probe cells in a near-native state, subject only to rapid cryo-preservation. Cryo X-ray iron fluorescence microscopy was used to quantify the amount of heme at different locations within the parasite. These fluorescence maps were overlaid on top of cryo soft X-ray tomography (SXT) images, which were used to visualize the parasite’s ultrastructure. The SXT images were also used to quantify the amount of hemoglobin in the parasite’s digestive vacuole.

Our results provide the first *in vivo* estimates of the heme crystallization rate in the malaria parasite, and also the first measurements of the amounts of non-crystalline heme present in the parasite’s digestive vacuole. We find that heme crystallization occurs at a rate consistent with catalysis by the heme detoxification protein^2^, which produces heme dimers, the building blocks of hemozoin. The measured rate is also close to the rate of synthetic hemozoin formation in buffer-saturated octanol^9^. In contrast, our measured rate is much faster than expected in either lipid or in aqueous phase. We also find that large amounts of non-crystalline heme are present in the digestive vacuole, and that the majority of this heme is most likely contained within whole or partially degraded hemoglobin. Thus we are led to an assembly line model in which hemoglobin degradation releases heme to the heme detoxification protein that assists formation of less toxic heme dimers, which then are released to form hemozoin crystals. Feedback must exist between digestion and crystallization such that digestion does not occur faster than dimer formation catalyzed by the heme detoxification protein. Regarding the final assembly-line step of hemozoin formation, we have earlier reported hemozoin crystals nucleated in an oriented manner at the inner leaflet of the digestive vacuole membrane^21–23^, indicating the role of lipid membranes as a template for accelerated nucleation of hemozoin from the dimers of heme. These findings have important implications for understanding the pathway of heme crystallization and for the development of new drugs against malaria.

## Results

### Correlative cryo soft X-ray tomography and cryo X-ray fluorescence microscopy

**Figure 1:**
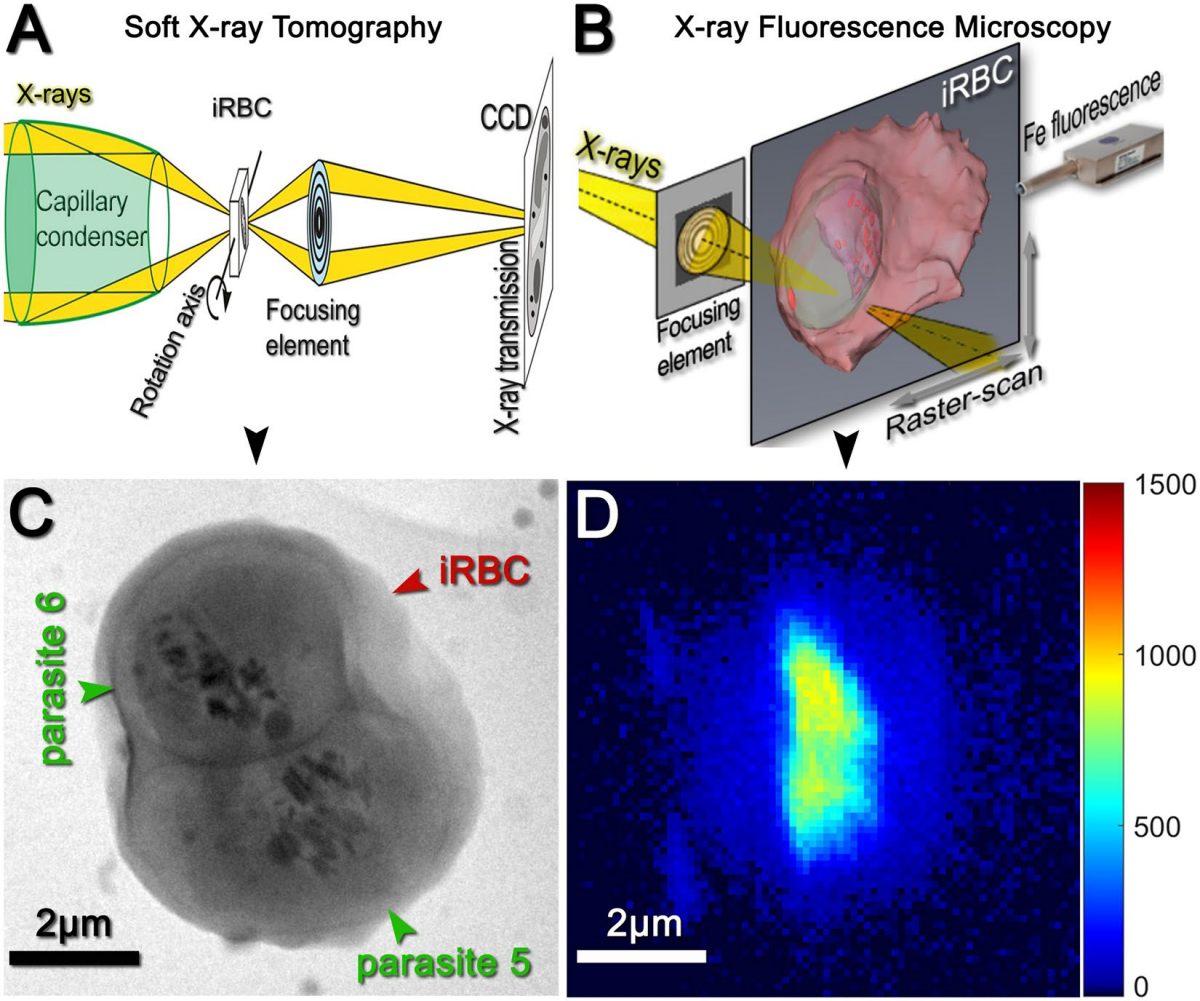
(**A**) Schematic view of soft X-ray tomography setup. (**B**) Schematic view of scanning X-ray fluorescence setup. (**C**) Non-aligned soft X-ray tomography image of a red blood cell (red arrow) infected with two malaria parasites (green arrows). (**D**) Non-aligned X-ray iron fluorescence map of the same cell obtained by raster scanning in X-ray fluorescence microscopy.

Our strategy to determine heme concentrations within the malaria parasite was to first find the location of both the parasite’s digestive vacuole and the hemozoin crystals within it using soft X-ray tomography (SXT, Fig. 1A) of cryo-preserved, parasite-infected red blood cells. Image contrast in SXT is based on absorption of soft X-rays by organic matter. This yields high contrast for carbon-dense structures, such as hemoglobin and other proteins, the hemozoin crystals, as well as all cellular membranes. In this way, SXT images enable identification of the red blood cell, the parasite within it, as well as the digestive vacuole and the hemozoin crystals within the digestive vacuole (Fig. 1C).

In order to measure iron concentrations at these different locations within the SXT image, we imaged the same cryo-preserved, parasite-infected red blood cell using hard X-ray fluorescence microscopy (XRF, Fig. 1B). We selected the X-ray energy in XRF to excite iron atoms, thereby producing an iron fluorescence map of the parasite and red blood cell (Fig. 1D).

The SXT and XRF images were collected on two different microscopes, so to examine iron concentrations at particular locations in the SXT image, the SXT and XRF images had to be aligned. We did this by correcting for the known axial tilt of the specimen on the XRF stage relative to the SXT stage, and also by accounting for the arbitrary in-plane rotation of the specimen grid as it was transferred between the microscopes (see Supplementary Information for the detailed calculation).

**Figure 2:**
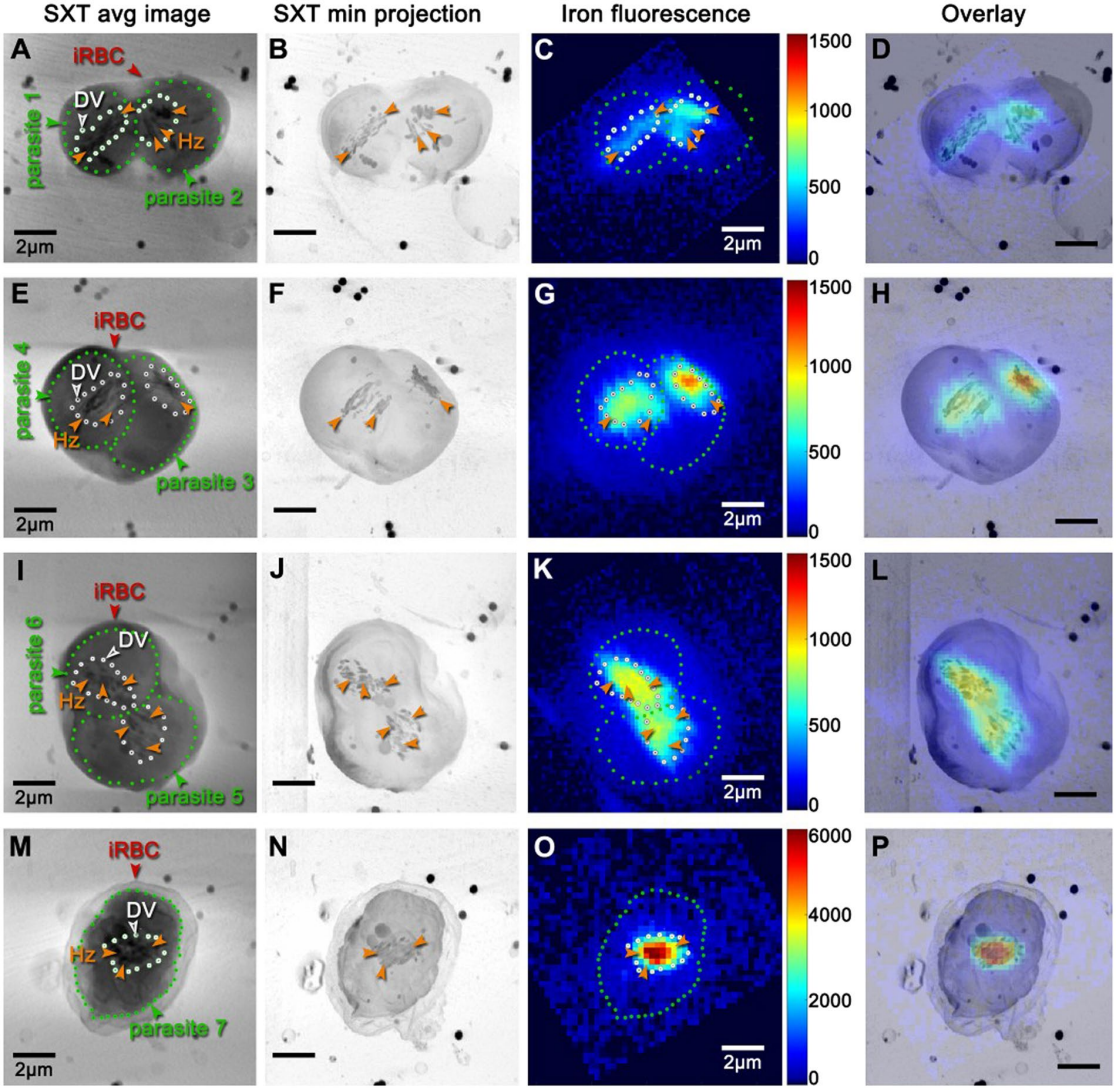
Correlative imaging of *Plasmodium* parasites by soft X-ray tomography (SXT) and scanning X-ray fluorescence (XRF). Shown are average intensity projections of four different soft X-ray tomography images (1^st^ column). Seven different parasites are visible (green arrows, dots and numbers) within four infected red blood cells (iRBCs) and red arrowheads). The digestive vacuole within the parasite (DV, white dots and arrowheads) can also be discerned, although precise determination of the DV boundary requires contrast adjustment of the images (Supplementary Fig. 1). Hemozoin crystals (Hz and orange arrowheads) are also visible as dark-grey spots due to strong X-ray absorption by their densely packed carbon content and iron. This high absorption enables clear detection of the crystals by computing a minimum projection of the 3D SXT images (2^nd^ column). We transferred these four iRBCs to the scanning XRF microscope and then collected images of iron fluorescence (3^rd^ column). Alignment of the SXT and XRF images was performed using the axial/in-plane alignment procedure described in the Results and Methods. This enabled transfer of the green and white dots indicating parasite and DV boundaries obtained from the SXT images to the fluorescence images. Finally, we generated overlays of the fluorescence and SXT minimum projection images (4^th^ column). Note that in these overlay images (4^th^ column) high iron fluorescence consistently overlays the Hz crystals detected by SXT. Note also that some background fluorescence can also be observed in the RBC cytosol surrounding the parasites (region between the white and the edge of the iRBC in **C,G,K**). This probably reflects undigested hemoglobin in the parasite. Consistent with this, very strong iron fluorescence within the DV correlates with very low iron fluorescence in RBC cytosol (**O**), suggesting that this parasite is at a later stage where most of the hemoglobin has already been digested and the heme converted into hemozoin crystals. Scale bar 2 μm.

The overlay images (Fig. 2) generated by this alignment for seven different parasites revealed three important features. First, we consistently observed the lowest iron fluorescence (blue, in Fig. 2, third column) in the regions overlaying the location of the red blood cells (the overlap is shown in Fig. 2, fourth column). This level of iron fluorescence corresponds to the amount of hemoglobin remaining in the red blood cell. Second, we consistently observed the highest levels of iron fluorescence in the regions of the parasite containing the hemozoin crystals. This fluorescence corresponds to the high levels of iron deposited into the crystals. Third, we also found that the amount of iron fluorescence associated with the crystals increased as a function of parasite age. In the oldest parasite (#7), for example, the iron fluorescence in the crystals was relatively high (Fig. 2O), and correspondingly the amount of fluorescence associated with the red blood cell was significantly lower, consistent with the conversion of the iron from hemoglobin in the red blood cell into hemozoin crystals within the parasite.

### Measurement of crystallized heme in the digestive vacuole

As a first step to estimate *in vivo* hemozoin crystallization rates we measured the total amount of heme present in the crystals within parasites of different ages. To ensure robust measurements, we developed three different complementary approaches to measure heme amounts.

#### Estimation of hemozoin heme content from the axial and in-plane alignment of the SXT and XRF images

The overlaid SXT and XRF images in Fig. 2 enable estimation of *in situ* iron levels in the hemozoin crystals by measuring the amount of fluorescence at their locations in the seven different parasite cells. We calibrated the fluorescence signal of the XRF by obtaining an image of an uninfected red blood cell, and then assigning the total fluorescence intensity from that red blood cell to the known amount of iron contained in a red blood cell (4 heme units times 2.7 × 10^8^ hemoglobin molecules^24^). With this calibration, we converted the fluorescent signal from the hemozoin crystals in each parasite of Fig. 2 to a total number of iron atoms within the crystals of that parasite (Table 1), based on the mathematical approach described in Kapishnikov *et al*.^25^.

**Table 1:** *In vivo* estimate of heme crystallization rate.

Parasite	#Fe (Hz) (×10^8^)	# nuclei	Stage # (# nuclei)	Age range [h]	Age ± esd [h]	Rate of Hz crystallization [heme/s]	Onset of Hz crystallization [h]
1	1.84	1 or 2	6 (1–2)	34–38	36 ± 1.6	6900 ± 2300	23.7 ± 0.7
2	2.92	1 or 2	6 (1–2)	34–38	36 ± 1.6		
3	3.02	1^(2)^	6 (1–2)	34–38	34 ± 1.9		
4	3.78	4	7 (3–5)	38–44	41 ± 2.4		
5	4.03	7	8 (6–~16)	44–48	44.4 ± 1.8		
6	4.37	5	7 (3–5)	38–44	44 ± 2.8		
7	7.67	≥12	8 (6–~16)	44–48	46.4 ± 1.6		
8	2.82	1	5 (1)	30–34	32 ± 1.6		
	3.02						
	3.03						

Shown in the 2^nd^ column are the total amounts of Fe atoms measured in the hemozoin crystals for the eight different parasites. The axial and in-plane alignment procedure was used to obtain the amounts of Fe for parasites 1–7, and for parasite 8 in its top row. The second Fe estimate for parasite 8 (middle row) was obtained from the 3D segmentation and fluorescent modeling alignment, and the third estimate (bottom row) from the volume of the crystals was obtained directly from the soft X-ray tomography (SXT) image. Note that the three different estimates for parasite 8 are close. The number of nuclei per parasite was obtained directly from the SXT image, and then converted to a stage based on published criteria^26^. Age was then estimated from this stage as described in Methods. Iron content vs. age were then plotted (Fig. 5), and the slope and y intercept of the best fitting line yielded the estimates for the heme crystallization rate and the onset of hemozoin (Hz) crystallization.

#### Estimation of hemozoin heme content from 3D segmentation and fluorescence modeling alignment

**Figure 3:**
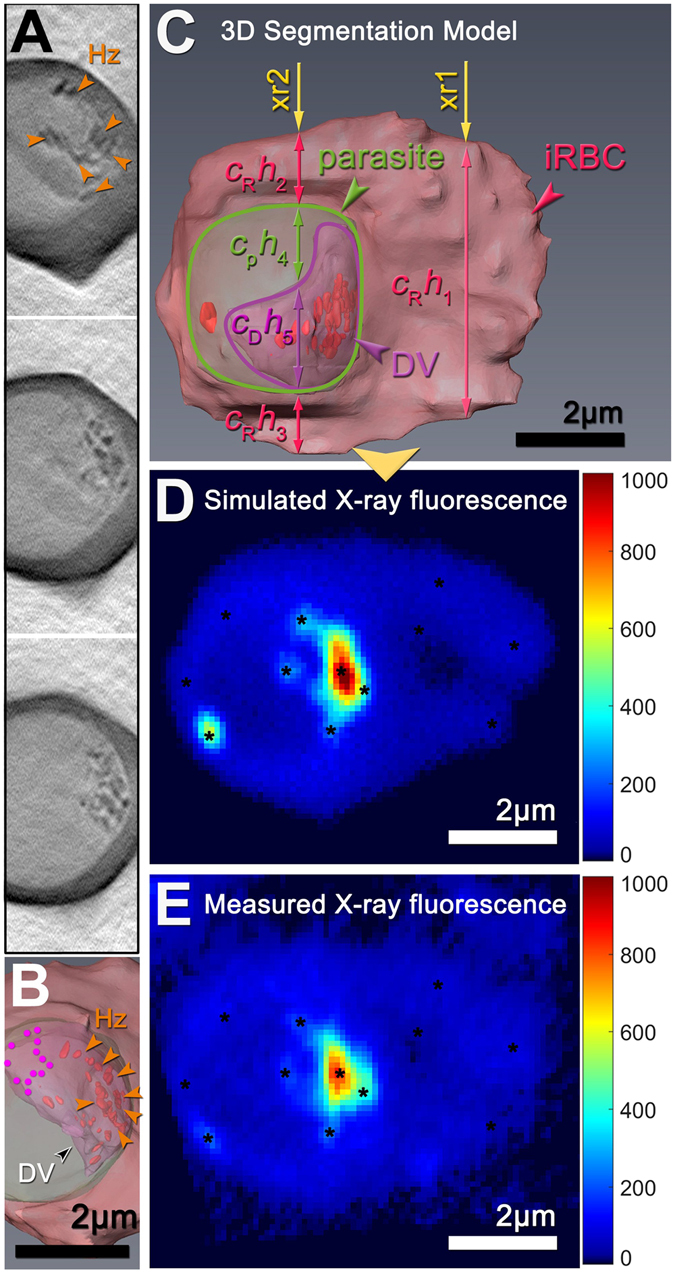
Alignment procedure by 3D segmentation and fluorescence modeling. Shown are selected Z slices from the SXT 3D reconstruction of parasite 8 and its red blood cell (**A**). Each slice in the 3D SXT image is hand segmented to identify the domains of key sub-structures, namely, the red blood cell (magenta), parasite (light green), digestive vacuole (purple) and hemozoin crystals (red) (**B**). The final 3D segmentation can then be used to predict the iron fluorescence obtained from an XRF scan, which produces a 2D projection of the iron levels in the red blood cell and parasite volume. Selected X-ray fluorescence paths (xr1 and xr2) through the specimen are shown (**C**). These traverse different domains of the specimen, which are of different thickness (*h*) and which contain different iron concentrations (*c*). Each domain therefore produces absorption proportional to the product *ch*, and the sum of these along any path is the predicted absorption in the XRF image. By assigning known iron concentrations to selected compartments, a predicted fluorescent image can therefore be generated (**D**, see Supplementary Information for a detailed explanation and calculation). The measured fluorescence image (**E**) can then be morphed onto the simulated fluorescence image, which will account for any random drift of the XRF stage during the measurement. In this way, the morphed XRF image can be overlaid onto the segmented 3D image to back calculate the amount of iron in the hemozoin crystals and the non-crystalline heme elsewhere in the digestive vacuole.

To account for the thermal drift of the XRF stage during the slow scanning acquisition of the iron fluorescence image, which effectively elongates the XRF image relative to the SXT image potentially corrupting overlay of the images, we developed a refined alignment procedure which corrected for arbitrary drift in the XRF image by overlaying it on a simulated fluorescence image obtained from a 3D segmentation of the SXT image. The overlay adjusted for arbitrary amounts of in-plane stretching in the XRF image by using an affine transformation between fiducial points in the two images (Fig. 3D,E).

**Figure 4:**
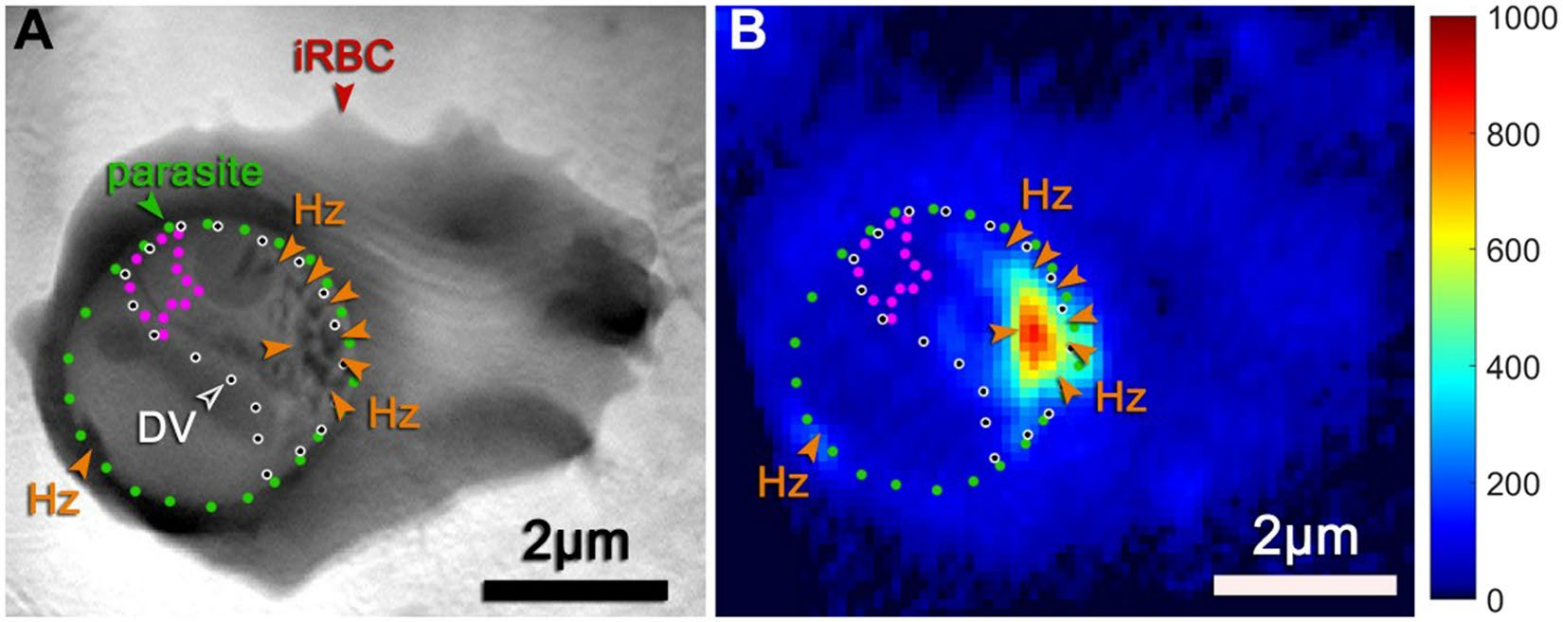
Infected red blood cell (parasite 8 in Table 1) imaged by soft X-ray tomography and scanning iron X-ray fluorescence and subject to alignment by 3D segmentation and fluorescent modeling as described in the Results and Methods. (**A**) Soft X-ray tomography projection image of the infected cell. The dotted lines delineate the parasite (green), its digestive vacuole (black-on-white), and the area of the digestive vacuole free from hemozoin fluorescence signal (magenta). Some of the hemozoin crystals are indicated by orange arrows. (**B**) Iron x-ray fluorescence map of the same cell aligned to its x-ray tomography dataset.

Another advantage of this refined alignment procedure is that it permits more precise measurement of iron in the crystals, since it allows for a direct calculation of the projection of the X-ray fluorescence signal through the 3D volume of the cell (Fig. 3). In this way, contributions from the red blood cell cytosol can be directly subtracted out leaving only the fluorescence arising directly from the crystals. Using this approach, we obtained an alignment of this cell (Fig. 4), and then determined the amount of iron in the hemozoin crystals of this aligned cell (~3.02 × 10^8^ atoms, Table 1, last row). This value was close to the value predicted for the same cell by the axial and in-plane rotation procedure described above (~2.82 × 10^8^, Table 1, last row), demonstrating that this simpler, first alignment procedure is reasonably accurate.

#### Estimation of hemozoin heme content directly from the SXT image

Finally, as a completely independent estimate for the number of heme monomers in the hemozoin crystals of the cell in Fig. 4, we also measured the total volume of the hemozoin crystals from the 3D SXT image of the cell. We then calculated the number of heme monomers present within this total crystal volume using the published crystal structure of hemozoin to determine the volume of a heme monomer (see Supplementary Information for detailed calculation). This yielded a second, non-fluorescence-based estimate for the iron content in the crystals (~3.03 × 10^8^ atoms, Table 1, last row) that was nearly identical to the fluorescence based estimate (~3.02 × 10^8^, Table 1, last row). This consistency among multiple measurements within the same cell demonstrates that our measurements of iron levels in the crystals are robust.

### Estimation of *in vivo* heme crystallization rates

**Figure 5:**
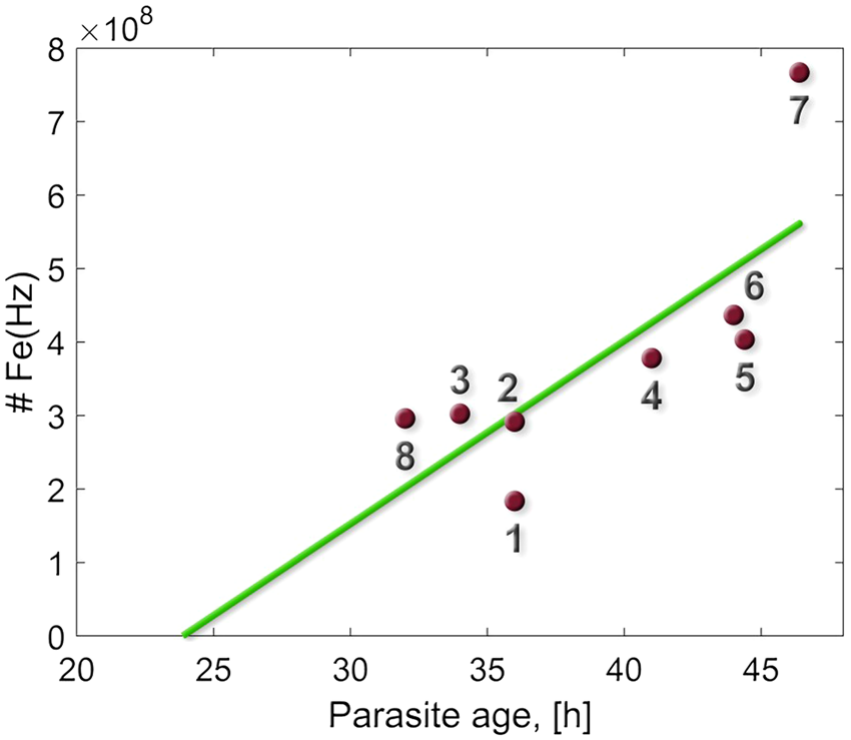
Hemozoin iron content as a function of the parasite age in parasites 1–8. Blue circles are the measured values taken from Table 1, and the green line is the fit with equal weights. The slope of the best fitting line yields the estimated rate of *in vivo* crystallization, and the *x* intercept the estimated onset time for heme crystallization.

We next estimated the age of each parasite in Fig. 2, using the procedure of Silamut *et al*.^26^, which is based on the number of nuclei and morphology of the parasite (Table 1). By plotting iron levels in the crystals as a function of parasite age (Fig. 5), we could then determine the rate at which iron, namely the heme dimer, was sequestered into the crystals. This plot showed a linear trend, with the slope of the best-fitting line being 6900 ± 2300 heme units/sec. At this rate, 72% of hemoglobin in the red blood cell^27^ would be consumed in about ${31}_{-8}^{+16}$ hours.

This slope therefore provides an estimate for the average *in vivo* rate of hemozoin crystal formation in these eight parasites. As an independent test of this fit, we examined the intercept of the best fitting line with the *x* axis, which should correspond to the average onset time of crystal formation. This intercept was ~24 hr., which is indeed consistent with estimates for when crystals can first be detected by light microscopy^26^. This consistency supports the validity of our *in vivo* rate estimate.

This *in vivo* estimate of heme crystallization rate can now be compared to published *in vitro* values for heme crystallization in an aqueous environment, in a lipid environment, in buffer saturated octanol, or catalyzed by the heme detoxification protein (Table 2). The *in vitro* values in Table 2 are derived from the published measurements and represent maximal possible crystallization rates. With these *in vitro* values for comparison, we find that our measured *in vivo* estimate for heme crystallization (~6900 heme units/sec) is more than two orders of magnitude faster than the *in vitro* rates in either water or in lipid, but agrees well with the measured *in vitro* rates in buffer saturated octanol (>3200 heme units/sec) and that catalyzed by the heme detoxification protein (~10,000 heme units/sec). Thus our measurements support the model of heme crystallization mediated by the heme detoxification protein, yet is also consistent with *in vitro* growth of synthetic hemozoin in buffer saturated octanol^2, 20^.

**Table 2:** Predicted *in vitro* rates of heme crystallization in various environments.

Heme crystallization environment	Rate [heme monomers/sec]
Aqueous environment alone (acetate medium)^16^	~0.05 (up to 0.15)
Lipid (purified MOG + acetate medium)^16^	~2.5 (up to 7.5)
Buffer saturated octanol^9^	>3200
Heme detoxification protein (HDP) (acetate medium)^2^	10^4^

The rates shown here were calculated based on published results^2, 9, 16^ (see SI for the detailed calculations).

### Measurement of non-crystalline heme in the digestive vacuole

To determine if this rate of heme crystallization was constrained by the amount of iron, as a reporter of heme, either contained in or delivered to the digestive vacuole, we next estimated how many non-crystalline heme molecules were present within the digestive vacuole. For this, we used the 3D segmentation of the cell in Fig. 4 which included a complete segmentation of the 3D volume of the digestive vacuole. Using the overlaid fluorescent image, we measured iron fluorescence within a sub-region of the vacuole (magenta region in Fig. 4B) that was sufficiently far away from the hemozoin crystals to preclude contamination of fluorescent blur from the crystals. This produced an estimate of the amount of non-crystalline heme in the sub-region. Using the volume of this sub-region, we find that the concentration of non-crystalline heme is 13 ± 8 mM. Given the reasonable assumption that the digestive vacuole is a freely diffusible space, this same concentration throughout the vacuole would result in 35 ± 22 × 10^6^ monomers of non-crystalline heme within the vacuole.

These measurements therefore show that there is a substantial amount of non-crystalline heme within the vacuole in this parasite, demonstrating that the heme crystallization rate is not limited by the amount of available iron within the digestive vacuole. Furthermore, given our estimated crystallization rate of ~6900 units/sec, the ~35 × 10^6^ monomers of non-crystalline heme remaining in this digestive vacuole would require ~1.4 hr. to crystallize. This large amount of non-crystallized heme cannot be free over this extended period, since free heme is instantaneously toxic^4^ and concentrations above 10 nM^28^ (corresponding to ~30 heme monomers given the volume of this digestive vacuole) cannot stay dissolved in water. Therefore our results predict the presence of another molecule in the digestive vacuole that is associated with the non-crystallized heme.

### Measurement of organic matter within the digestive vacuole

The simplest model is that the non-crystalline heme in the digestive vacuole is associated with yet to be digested hemoglobin. It is known that a hemoglobin molecule contains four heme monomers, so the ~13 mM concentration of non-crystalline heme in the digestive vacuole would require ~3.2 mM of hemoglobin. This high concentration of hemoglobin is plausible since the red blood cell contains ~5 mM hemoglobin.

**Figure 6:**
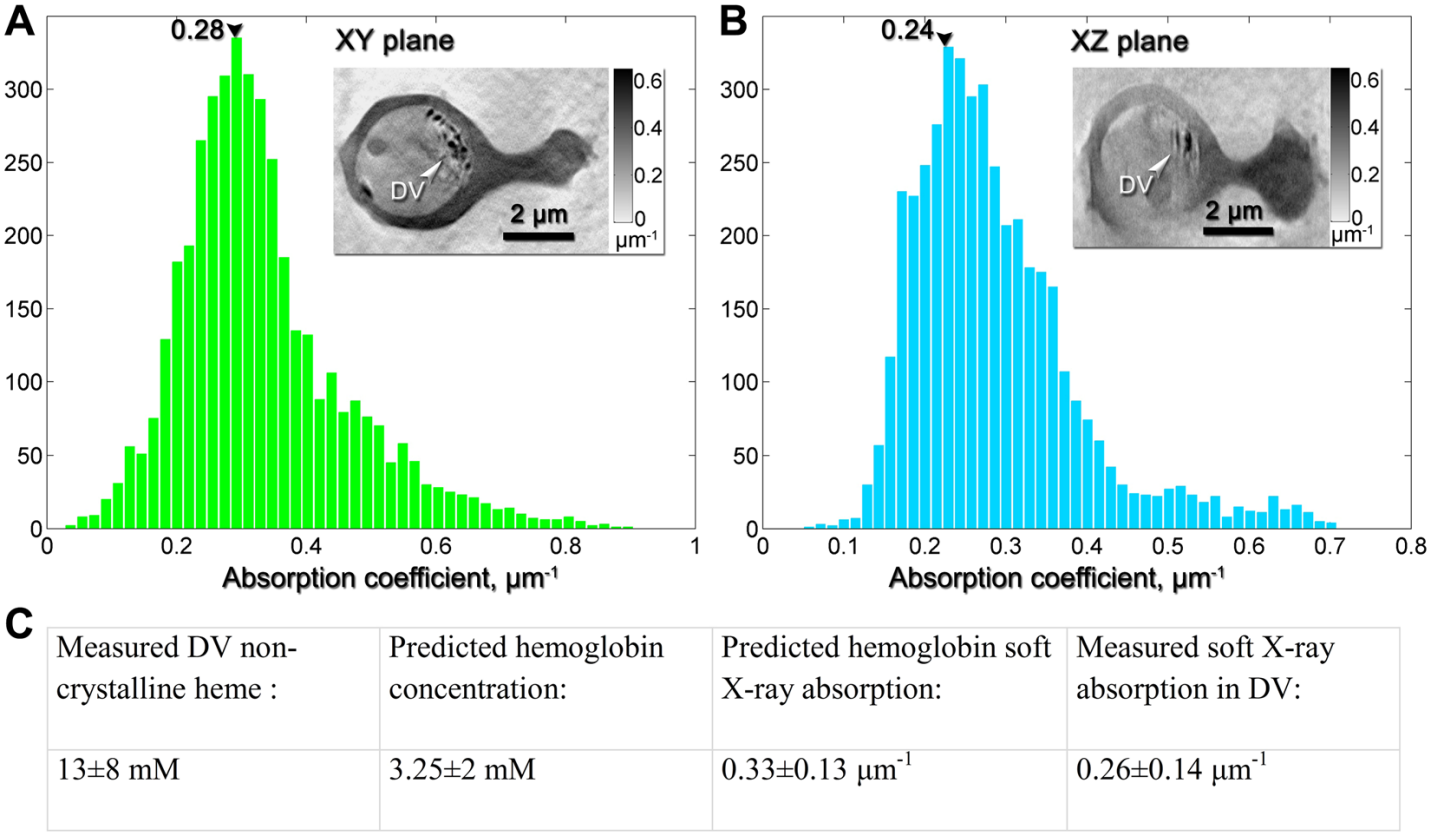
Measured X-ray linear absorption coefficients within the parasitic digestive vacuole, derived from the grey scale intensity contrast of the SXT data. (**A**) Absorption coefficient distribution in the digestive vacuole (DV) measured in a tomographic slice in the XY plane, which is parallel to the zero-tilt SXT projection, perpendicular to the optical axis Z and in a plane similar to that of the Fe fluorescence map. (**B**) Measurement in the XZ plane, which is parallel to the optical axis and perpendicular to the XY plane. Insets: corresponding grey scale images of the tomographic slices in the XY and XZ planes. The absorption coefficients are derived from the pixels within the digestive vacuole (DV). The histograms peak at 0.28 and 0.24 μm^−1^, and the mean of these values and the mean of their standard deviations yield 0.26 ± 0.14 μm^−1^. (**C**) This measured absorption is then compared to the predicted absorption for hemoglobin in the digestive vacuole, as would be required to store the 13 mM heme predicted from XRF measurements.

Such a large amount of hemoglobin should be detectable in the SXT image of the vacuole, since image contrast in SXT arises from the presence of organic matter. This is a quantitative relationship, since the amount of X-ray absorption expected at a specific energy can be directly calculated based on the molecular formula of the absorbing molecules and their predicted concentrations. Therefore, we calculated the amount of absorption expected from ~3.2 mM hemoglobin dissolved in water within the digestive vacuole using established procedures^22^ to obtain an estimate of 0.33 ± 0.13 μm^−1^ (Fig. 6).

To compare this prediction to our images, we measured the greyscale values in the SXT images of the digestive vacuole by using the central XY and XZ slices of the 3D image. The greyscale voxel (volumetric pixel) values of the 3D reconstructed image of the parasite 8 scale linearly with the actual X-ray absorption coefficients within the sample. This linear relationship can be explicitly determined by using two known chemical compositions within the sample, in our case first the surrounding ice and second the hemoglobin concentration in the red blood cell cytoplasm (see Methods). From this we could convert the intensity of each pixel in the central XY and XZ slices of the parasite’s digestive vacuole into absorption coefficients, yielding histogram plots of absorption coefficients (Fig. 6). This yielded a mean absorption coefficient of ~0.26 ± 0.14 μm^−1^ in the digestive vacuole, which is within error of the predicted value of 0.33 ± 0.13 μm^−1^ for 3.2 mM hemoglobin in the digestive vacuole. These results therefore suggest that the non-crystalline heme in the digestive vacuole is most likely associated with undigested hemoglobin, and therefore imply that hemoglobin digestion must be tightly controlled in order that it does not exceed our measured *in vivo* rate of heme crystallization.

## Discussion

It is generally accepted that the primary route for heme detoxification in the malaria parasite is via crystallization into hemozoin crystals. However, recent studies demonstrating heme transport proteins in the malaria parasite^5^ have renewed interest in the possibility that heme is also detoxified by export from the parasite cytoplasm. Using our measurements of heme levels in the crystals, we now find that the oldest parasite that we examined contains about 7.67 × 10^8^ iron in its crystals, that is 70% of the total red blood cell iron. This suggests that heme crystallization is indeed the primary pathway for detoxification and impeding crystal growth seems to be an intelligent strategy for drug development.

The model that we shall present for hemozoin formation is based on the present data and our earlier work together with several published papers. We maintain that heme monomers are dimerized prior to oriented crystal nucleation at the membrane surface of the digestive vacuole^21–23^ followed by its growth. This statement is supported by the stereochemical and lattice complementarity of diacylglycerol, and/or perhaps other lipid head groups, and the {100} faces of Hz crystals which we demonstrated in Kapishnikov *et al*.^23^. The dimerization process is catalytically induced by the protein HDP as reported by Jani *et al*.^2^, Chugh *et al*.^6^, and Ishikawa and coworkers^20^. We note that, the heme monomer is highly reactive whereas the dimer is almost inert since its Fe is less accessible to the external environment as it is shifted by 0.6 Å towards the center of the dimer.

The alternative mechanism of hemozoin nucleation is lipid-mediated. Recently, Kuter *et al*.^19^ have described a detailed model (*cf*. their Fig. 7) that involves heme partitioning from an aqueous medium into lipid environment. This step is followed by oriented assembly of the heme molecules within the lipid medium leading to Fe-O(propionate) coordination to form the cyclic heme dimers and the natural propensity for the heme dimers to interlink via O-H^…^O bonds. The subsequent growth of hemozoin crystals follows the vapor-liquid-solid crystallization mechanisms described by Vekilov *et al*.^29^.

**Figure 7:**
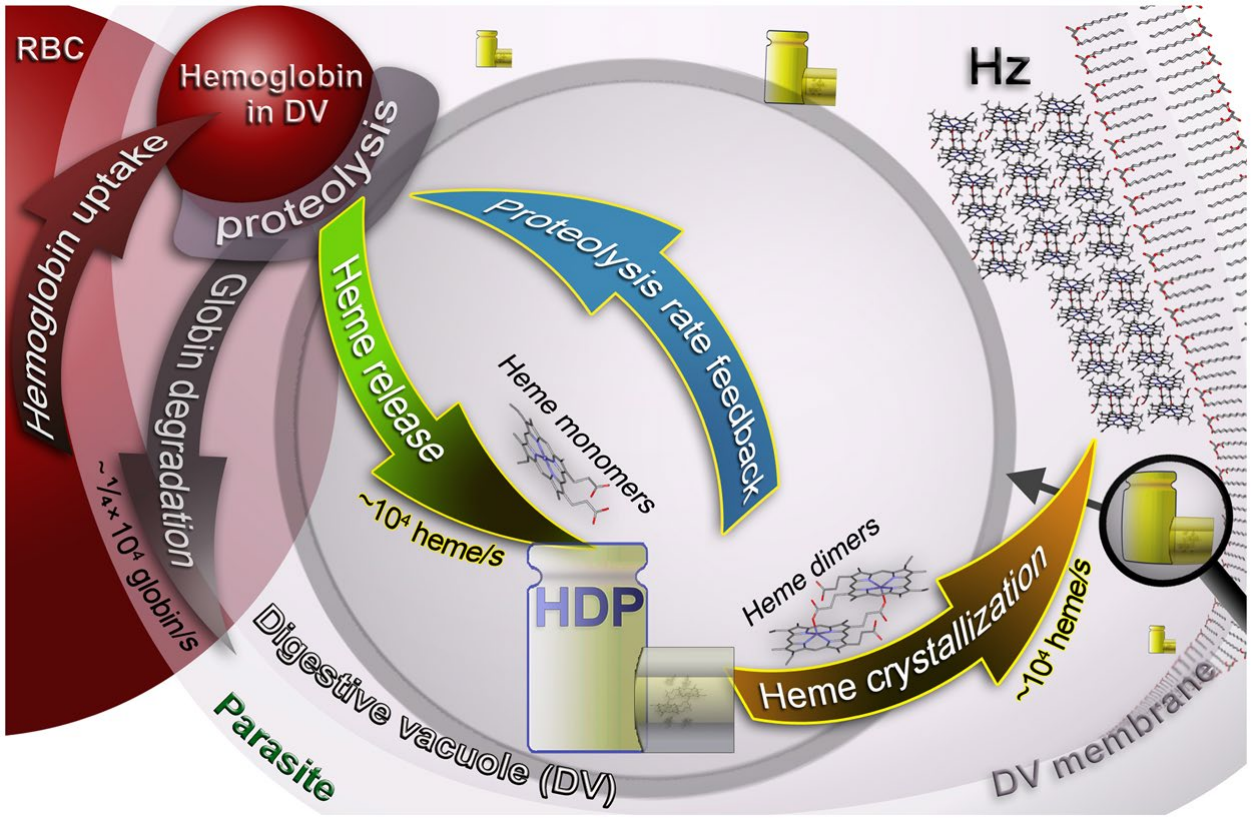
Assembly line model for hemozoin (Hz) crystal formation in the digestive vacuole of a RBC infected by the malaria parasite *P. falciparum*, in the trophozoite stage. Our measurements show that *in vivo* heme crystallization occurs at a rate of ~10^4^ s^−1^ (brown arrow). This rate is close to the *in vitro* rate of heme dimerization measured for the heme detoxification protein (HDP), and so we propose that HDP is primarily responsible for heme crystallization. Our measurements also show that there is considerable non-crystalline heme in the digestive vacuole and that this heme is most likely contained within hemoglobin (small red sphere at top). Thus in order to avoid overproduction of heme monomers and dimers, hemoglobin digestion and heme dimerization must proceed at a rate (green arrow) equal to the rate of heme crystallization. As a result, we propose a feedback mechanism (blue arrow) between crystallization and proteolysis to control the rates of proteolysis. We propose that the heme dimers produced by HDP either bind to existing hemozoin crystals or form new crystals via heterogeneous nucleation in an aqueous environment at the inner leaflet of the digestive vacuole membrane^22, 23^. With each hemoglobin molecule containing four heme monomers the rate of globin degradation (grey arrow) is ¼ that of heme monomer release.

This model of hemozoin nucleation and growth in lipid environment requires hemozoin crystals engulfed in lipid medium. SXT microscopy provides direct native contrast image of a biological cell subject only to vitrification with no need for any chemical treatment. At the X-ray energy used for the SXT data collection (*cf*. Methods) the absorption coefficient of a lipid medium is approximately ten times higher than that of water making lipid structures directly observable (see cellular ultrastructure in Supplementary Fig. 3). At the measured half-pitch resolution of 31 nm (for 40 nm ZP objective) and 26 nm (for 25 nm ZP objective) a “shroud” of lipid surrounding hemozoin crystals has not yet been detected (see Supplementary Fig. 3). A lipid shroud surrounding hemozoin crystals would have to be thinner than 26 nm in order to remain undetected.

Our data has also yielded a measure of the *in vivo* rate of heme crystallization by the parasite, making use of the parasite’s age based on its ultrastructure. This calculation yielded an estimate of 6900 ± 2300 heme units/sec. Strikingly, this *in vivo* crystallization rate is similar to the measured *in vitro* rate of heme dimerization by the heme detoxification protein (HDP). Our measured rate also matches the rate of growth in buffer saturated octanol^9^ indicating an optimal growth rate mechanism irrespective of the environment; however, for the reasons given above, we presently favor the HDP model.

Using XRF we were also able to derive the amount of non-crystalline heme within the digestive vacuole, which was measured from the total fluorescence within the parasite’s digestive vacuole excluding the fluorescence associated with the crystals. This produced a content of 35 ± 22 × 10^6^ heme monomers corresponding to a concentration of ~13 mM heme, an amount of iron that would require 1.4 hr to crystallize based on our ~6900 heme units/sec *in situ* crystallization rate. These results demonstrate first that the rate of heme crystallization is not limited by the amount of (not-yet-crystallized) heme present in the parasite’s digestive vacuole, and second that this non-crystalline heme must be associated with some other molecule in the digestive vacuole since free heme at a mM concentration would be highly toxic to the parasite.

We then tested the simple model that the large amount of non-crystallized heme was still incorporated within undigested hemoglobin. We found that the amount of absorption contrast within the digestive vacuole determined by SXT was consistent with the amount of hemoglobin required to store the amount of heme monomers determined by XRF. Alternative possibilities include that the majority of heme is associated with another protein other than hemoglobin or with lipid droplets. The former may be ruled out since our measurements show that mM protein levels are required to store the measured amount of heme, and only hemoglobin is likely to be present at such high concentrations. The latter is also unlikely for we did not detect lipid bodies within the digestive vacuole^22^, even though they are easily visible in other cell types by SXT (unpublished observations). Furthermore, based on previous studies of heme solubility in an octanol solution (mimicking lipid)^28^, much higher concentrations of lipid would be required compared to protein in order to store the measured amount of non-crystallized heme. This would then give rise to a much higher absorption contrast in the digestive vacuole than we measured (see Supplementary Information for calculations). At this point, it is noteworthy that in a separate study^25^ we describe measurements of X-ray fluorescence signals obtained from S and Fe atoms in pristine RBCs yielding an atomic ratio close to 3:1 which matches the 3:1 molar ratio of S:Fe in hemoglobin. Fluorescence measurements of *Plasmodium* infected RBCs have revealed the presence of S in the various organelles indicating that in the future it should be possible to determine the relative amount of heme still part of hemoglobin and ‘free’ heme still not part of hemozoin in the DV.

Together our observations lead us to propose a new assembly line model for heme detoxification in the digestive vacuole (Fig. 7). Our data suggest that non-crystalline heme is stored within hemoglobin in the digestive vacuole, and then converted to crystallized heme with the assistance of the heme detoxification protein. If the digestion of hemoglobin would proceed at a rate faster than our measured crystallization rate of 6900 ± 2300, namely faster than the rate of heme dimerization by HDP, the concentrations of free heme within the digestive vacuole would quickly rise to toxic levels. According to an analysis of the results reported by Chugh *et al*.^6^ (see Supplementary Information) the potential rate of hemoglobin degradation by falcipain 2 protease is much higher than the measured rate of hemozoin crystal growth. This analysis leads us to suggest that some form of coupling exists between the rate of hemoglobin digestion and the rate of heme dimerization and crystallization, perhaps realized via an association of falcipain 2 protease and HDP^6^. If this coupling is in any way disrupted, free heme levels would rise and kill the parasite.

We are not in the position to suggest a specific molecular model for this coupling but we trust that our proposed assembly model, depicted in full in Fig. 7, can inspire future biochemical research as our assembly line model suggests that the molecules involved in coupling hemoglobin digestion with heme dimerization are key targets for drug disruption in the parasite.

## Methods

### Parasite culture

The *Plasmodium falciparum* parasite culture (3D7 strain) was grown in flasks at 37 °C with human serum supplied by Haema (parasites 1–7) or, alternatively, artificial human serum Albumax II, Invitrogen (parasite 8), all following established protocols^30^. Following centrifugation of the parasite culture, the supernatant was removed and the pellet was re-suspended in PBS at ~1:1 volume ratio. This concentrated parasite sample was then transferred onto copper grids for freezing.

### Sample preparation for X-ray microscopy

Prior to freezing, a small volume of ~350 nm diameter polystyrene beads in a citric buffer solution was added to the concentrated parasite sample to serve as fiducial markers for tomographic reconstruction. A microliter quantity of the concentrated parasite sample with beads was then spread on a specimen carrier grid and vitrified by high pressure freezing^31, 32^. We used Ø3mm round TEM finder grids (2SPI) for parasites 1–7 and a rectangular IFR-1 grid (Gilder Grids) for parasite 8.

### X-ray fluorescence microscopy

X-ray fluorescence maps were collected at the microXAS beamline, Swiss Light Source. The X-ray beam of energy of 7.2 keV was focused with a kinoform lens^33^ to a spot size of 400 × 300 nm^2^. The sample was moved in plane across the nanofocused beam in steps of 100 nm (parasites 1–6, 8) and 200 nm (parasite 7) creating a raster scan. The X-ray fluorescence signal was collected with a Ketek detector with 1 sec (parasites 1–6), 0.2 sec (parasite 7) and 0.5 sec (parasite 8) acquisition times. In order to avoid shadowing of the fluorescence signal by the grid bars, the sample was rotated around its vertical axis by 10° towards the detector. The sample temperature was maintained at −170 °C by a stream of cold nitrogen using CryoJet 3, Oxford Systems.

### Soft X-ray tomography

The soft X-ray tomography (SXT) data for parasites 1–7 were collected at a full-field transmission X-ray microscope at the MISTRAL beamline^34^, ALBA Synchrotron Light Source. The X-ray beam energy was 520 eV. A 40 nm zone plate was used as an X-ray objective. The image pixel size was 13 nm. Each sample was imaged over a range of 131 to 141 tilt angles with 1° step size.

SXT data for parasite 8 were collected at a full-field transmission X-ray microscope at the U41-XM beamline^35^, BESSY-II synchrotron, with X-ray energy of 510 eV, and a 25 nm zone plate. The image pixel size was 10 nm. Parasite 8 was imaged over a range of 111 tilt angles with 1° step size.

### Correlating the XRF and SXT images

The two different procedures used to align the XRF and SXT images have been described in the Results section. Step-by-step protocols with example calculations are provided in the Supplementary Information.

### Calculation of iron levels

The procedure to convert iron fluorescence into iron concentrations has been described in the Results. See the Supplementary Information for explicit calculations of the conversion coefficient. This conversion coefficient was then used to determine iron concentrations at different locations in the parasite. However, since the iron concentration obtained by XRF is a two-dimensional projection through the cell (Fig. 3C), we needed to subtract out the contributions to this fluorescence measurement from iron contained in regions above or below the region of interest. Specifically, for measurement of total non-crystalline heme in the digestive vacuole we subtracted out the contribution of iron from the red blood cell, and for measurement of total iron within the hemozoin crystals, we subtracted out the estimated contributions from both non-crystalline heme within the digestive vacuole and from hemoglobin within the red blood cell. The explicit calculations for this subtraction procedure are given in the Supplementary Information for each of the two alignment procedures used.

### Measurement of absorption coefficients in digestive vacuole of cell 8

The absorption of X-rays in the specimen is subject to the Beer-Lambert law allowing quantification of concentrations. For tomographic reconstruction, this law states that absorption coefficients in the 3D specimen *μ*(*i, j, k*) are linearly proportional to grey scale values *M*(*i, j, k*) in the SXT image^36^. To find the equation of this line relating grey scale values to absorption, two points are needed. For one point, we measured the intensity values in the ice around the specimen assigning the known absorption of ice (*μ*_ice_ = 0.11 μm^−1^). For the other point, we measured the intensity values in the red blood cell assigning the calculated absorption coefficient for 5.5 mM hemoglobin in water (*μ*_Hgb_ = 0.45 μm^−1^) (Kapishnikov *et al*.^22^). Having defined the slope and *y* intercept of this line, we then used it to convert the average intensities within the digestive vacuole of a specimen into an average absorption coefficient. This average absorption was then used to calculate the concentration of hemoglobin and water in the digestive vacuole based on the molecular formulas for water and hemoglobin (Kapishnikov *et al*.^22^).

### Estimation of parasite age

Silamut *et al*.^26^ report age ranges for post-invasion parasites along with the number of parasite nuclei present in the red blood cell (see their Fig. 1, where the size ratio of the parasite and the host cell can also be roughly evaluated). Based on the abovementioned features, parasites 1–8 were assigned to one of the four following age ranges in Silamut *et al*. defined in their Fig. 1 (columns #5–8): column (#5) 1 nucleus, 30–34 hours post-invasion, column (#6) 1–2 nuclei, 34–38 hours post-invasion, column (#7) 3–5 nuclei, 38–44 hours post-invasion, column (#8) 6–~16 nuclei^26, 37^, 44–48 hours post-invasion. Within a selected range the age of the parasite was calculated based on its own number of nuclei assuming that within a single range the parasite age is linearly proportional to the number of nuclei given at the range margins. 30–34 is an exception to this assumption since the entire range is given for one nucleus, therefore in this case we assigned the mean of the range as the parasite age. Since parasite 8 has only one nucleus, we used its volume to distinguish between ranges 30–34 and 34–38.

## Electronic supplementary material

Supplementary information
