# Supplementary material for: Unraveling heme detoxification in the malaria parasite by in situ correlative X-ray fluorescence microscopy and soft X-ray tomography
Source: Sci Rep. 2017 Aug 8;7:7610. doi: 10.1038/s41598-017-06650-w (PMC5548722; doi:10.1038/s41598-017-06650-w)
Supplement: Supplementary file 1 — Supplementary information [file 41598_2017_6650_MOESM1_ESM.pdf]

**Unraveling heme detoxification in the malaria parasite by *in situ* correlative X-ray fluorescence microscopy and soft X-ray tomography**

Sergey Kapishnikov[1,2*], Daniel Grolimund[3], Gerd Schneider[1,4], Eva Pereiro[5], James G. McNally[1*], Jens Als-Nielsen[2*] and Leslie Leiserowitz[6*]

[1] Soft Matter and Functional Materials, Helmholtz-Zentrum Berlin, Albert-Einstein-Str. 15, D-12489 Berlin, Germany

[2] Niels Bohr Institute, University of Copenhagen, Universitetsparken 5, 2100 Copenhagen, Denmark

[3] Paul Scherrer Institute, 5232 Villigen PSI, Switzerland

[4] Institute of Physics, Humboldt University, Newton str. 15, 12489 Berlin, Germany

[5]ALBA Synchrotron Light Source, MISTRAL Beamline−Experiments Division, 08290 Cerdanyola del Valles, Barcelona, Spain

[6] Dept of Materials and Interfaces, Weizmann Institute of Science, Rehovot 76100, Israel

## Supplementary information

### Conversion of X-ray iron fluorescence signal to iron content

To convert the X-ray iron fluorescence signal to iron quantity we divided an established value of $4 \times 270 \times 10^6$ iron atoms in human red blood cells [1] by the total iron fluorescence count in an uninfected red blood cell (for parasites #1-7), or by the total iron fluorescence count of the infected red blood cell itself (for parasite #8, which was mapped during another scheduled time slot and different microscope alignment) yielding a coefficient that converts X-ray iron fluorescence to the iron quantity. In both cases the conversion coefficient was similar, 3600 and 3900 respectively.

### Alignment of soft X-ray tomography and X-ray fluorescence images

Alignment Method 1 – Simple alignment based on axial tilt and grid bar rotation for parasites #1-7.

The relative in-plane rotation of the specimen in the transmission X-ray microscope used for collection of soft X-ray tomography (SXT) images and the scanning X-ray fluorescence setup (XRF) was estimated from the orientation of the grid bars imaged in both cases (see Supplementary Fig. 2). We determined the relative in-plane rotation by comparing the orientation of the letter “E “printed on the specimen grid and the grid bars shown in Supplementary Fig. 2. The specimen grid in-plane rotation in the transmission X-ray microscope

microscope was determined as 224° counter-clockwise (Supplementary Fig. 2 left), and it was not tilted around its vertical axis. When mapped by XRF, the same grid was rotated in-plane by ~6° counter-clockwise (Supplementary Fig. 2 right) and also tilted by 10° around its new vertical axis toward the fluorescence detector, i.e. the axial tilt of the XRF image. Therefore, the difference in specimen in-plane rotation during SXT and XRF data collection was ~218°.

To align both datasets, the SXT 3D reconstructed image was rotated by ~218° clockwise and then by -10° rotation around its new vertical axis.

Alignment Method 2 – Modeling of iron distribution and affine transformation to measured data used in parasite #8.

This method starts with the simple alignment (Method 1) followed by a further refinement described in the next paragraph. The in-plane orientation of the sample grid (IFR-1) in SXT and the XRF microscopes differed 90°. The grid tilt around its vertical axis in the XRF setup was 8°. Hence we rotated the 3D segmentation by 90° clockwise in-plane and then by -8° around its new vertical axis.

Following this simple alignment, we simulated the X-ray fluorescence map by assigning iron densities to the 3D volumes of the hemozoin crystals (1.42 atoms/nm$^3$ known from their crystal structure [2]) and the red blood cell cytosol (~0.012 atoms/nm$^3$, corresponding to 5mM hemoglobin concentration in a normal red blood cell [3]). The resulting 3D map of iron was projected along the axis of the x-ray beam resulting in a 2D map of iron content (Fig. 4A). The iron quantity was converted to fluorescence counts using the fluorescence conversion coefficient. The resulting fluorescence image was convolved with a 2D Gaussian function and Poisson noise was added (Fig. 4B) to simulate the actual measurement (Fig. 4C). The close resemblance of the simulated and the experimental maps allowed selection of twelve fiducial points on each of them (black stars on Fig. 4 B and C). An affine transformation of the fiducial point coordinates in both maps was calculated using the *cp2tform* function from the Image Processing Toolbox, MATLAB. The resulting transformation was then applied to the fluorescent map, thereby compensating for thermal sample drift during the fluorescence scan.

**Estimate of iron concentration in the parasite compartments**

The iron maps obtained by the X-ray fluorescence scans are two dimensional projections of different iron containing structures with various iron concentrations. The different paths of x-rays through the cellular compartments with various concentrations of iron give variation in the projected iron content. For instance, consider two X-ray beam paths xr1 and xr2 shown in the main text Fig. 3C. In the xr1 case, the X-ray beam illuminates only the red blood cell cytosol, hence the resulting projected iron content is given by a product of iron density $c_R$ and the beam path $h_1$, multiplied by the pixel area $A$, i.e. $c_R h_1 \times A$. In the xr2 case, the total iron count in the corresponding pixel is a sum of the products of iron densities and x-ray beam paths in the

cellular compartments it traverses multiplied by the pixel area, $(c_R(h_2+h_3)+c_p h_4+c_D h_5)\times A$. Subscript R corresponds to the red blood cell cytosol, p to the parasite cytosol, D – the digestive vacuole excluding the hemozoin crystals

We used two methods to identify the iron content in hemozoin crystals and the non-crystalline heme concentration in the digestive vacuole.

Method 1 – Estimate of iron content in hemozoin crystals from XRF data in cells 1 – 7

This is a first approximation to iron concentrations based on the simpler alignment procedure (Method 1) described above. The iron density in the hemozoin crystals is 1.42 atoms/nm$^3$. For comparison, the iron density in 5mM RBC's hemoglobin is 0.012 atoms/nm$^3$. Hence, hemozoin crystal content yields high iron fluorescence signal, notably higher than that of the rest of the cellular content (Fig.2).

Following the alignment procedure, we verified that the area of the high iron fluorescence signal matches the location of hemozoin crystals in the SXT images. Using the fluorescence conversion coefficient $k_{\text{cts}\rightarrow\text{Fe}}$=3600 (*vide supra*), we converted the iron fluorescence signal to iron content. We then measured the projected iron content near (but outside) the area containing hemozoin crystals, at a distance of 300-600 nm away from the edge of the sharp increase in the iron signal due to the crystals. Such a measurement should contain non-crystalline iron content, $\text{Fe}_{\text{meas}}(i_0,j_0) = \text{Fe}_{\text{R+p+D-Hz}}(i_0,j_0)$, where $i_0,j_0$ are pixel coordinates near (but outside) the hemozoin-containing area, with subscript D–Hz denoting the digestive vacuole excluding the hemozoin crystals. This non-crystalline iron is found in the red blood cell cytosol, the parasite cytosol, and as non-crystalline heme within the digestive vacuole. Conversely, the area of high iron counts includes both non-crystalline and crystalline iron, i.e. $\text{Fe}_{\text{meas}}(i_1,j_1) = \text{Fe}_{\text{R+p+D-Hz}}(i_1,j_1) + \text{Fe}_{\text{Hz}}(i_1,j_1)$ where $i_1,j_1$ are pixel coordinates inside the Hz-containing area. We make a reasonable assumption in this measurement that over a small area the variation of the projected non-crystalline iron content, $\text{Fe}_{\text{R+p+D-Hz}}(i,j)$, is insignificant. Thus the average level of the projected non-crystalline iron content near and within the relatively small Hz-containing area should be similar:
$\langle \text{Fe}_{\text{R+p+D-Hz}}(i_0,j_0)\rangle \approx \langle \text{Fe}_{\text{R+p+D-Hz}}(i_1,j_1)\rangle$

Thus we can estimate hemozoin iron content by simple subtraction of the average of non-crystalline iron content from the surrounding area:

$$\text{Fe}_{\text{Hz}} = \sum_{i_1,j_1} [\text{Fe}_{\text{meas}}(i_1,j_1) - \langle \text{Fe}_{\text{meas}}(i_0,j_0)\rangle]$$

Method 2 – Estimate of iron content in cell 8 from XRF data and aligned volumes from SXT data.

This method required precise alignment of the XRF map and the SXT segmented volumes since it relies on precise measurement of the X-ray beam path through the cellular compartments.

Consider two x-ray beam paths, xr1 when the X-ray beam passes though one cellular compartment and xr2, when the X-ray beam passes through three cellular compartments Fig. 4A:
**xr1 path** - In the case of the xr1 path, the beam traverses one cellular compartment, namely red blood cell cytosol. The iron content in this case is given by the formula $c_R h_1 \times A$, where $c_R$ is iron concentration within the red blood cell cytosol, $h_1$ is the x-ray beam path through the cytosol and $A$ is the pixel area. The cytosol volume corresponding to this pixel ("projected volume") is $h_1 \times A$ a product of the pixel area and the x-ray beam path length through this compartment. The iron concentration along this path can be easily calculated by division of the XRF iron count by the SXT projected volume.
**xr2 path**- The iron signal along this path is a sum of products of iron concentrations along each of the cellular compartments and their corresponding volumes, i.e. $(c_R(h_2+h_3)+c_p h_4+c_D h_5)\times A$. In this case the calculation of iron concentration in a compartment of interest depends on knowledge of iron concentrations in the remaining compartments. Thus, in order to calculate iron concentration in, say, the digestive vacuole (DV) we should first measure iron content in an area where the X-ray beam traverses only the red blood cell cytosol that will yield $c_R$. With known $c_{R,}$ then we should measure iron content in the area of two compartments, e.g. the red blood cell cytosol and the parasite. This will yield $c_P$. Knowing $c_P$ and $c_R$, we then need to measure iron content in the area of three compartments: the DV, the parasite cytosol and the red blood cell cytosol. This way we will calculate $c_D$ since the iron concentration in the other compartments are obtained in the two previous steps.

Due to the presence of the hemozoin crystals, the iron concentration within the DV is non-homogeneous. We can measure the iron concentration in the DV in every pixel of the projected digestive vacuole area $c_{\mathrm{D}}(i,j)$. The average of this value multiplied by the volume of the DV yields the total iron content:
$\mathrm{Fe_{total}(DV)} = \langle c_{\mathrm{D}}(i,j)\rangle \cdot V_{\mathrm{DV}}$

Measurement of $c_{\mathrm{D}}(i_0,j_0)$ within the DV but away from the hemozoin crystals (see area outlined with purple dots in Fig.3) gives the concentration of non-crystalline iron (i.e. non-crystalline heme), $c_{\mathrm{D-Hz}}(i_0,j_0)$. See Supplementary Table 1 for the concentration values. That, multiplied by the volume of the DV excluding that occupied by the crystals gives the total content of non-crystalline iron: $\mathrm{Fe_{non\text{-}Hz}(DV)} = c_{\mathrm{D-Hz}}(i_0,j_0) \cdot V_{DV-Hz}$

The iron content of hemozoin crystals can then be calculated as
$\mathrm{Fe_{Hz}(DV)} = \mathrm{Fe_{total}(DV)} - \mathrm{Fe_{non\text{-}Hz}(DV)}$

**Estimation of iron content in hemozoin crystals of cell 8 using the SXT data alone**

Hemozoin crystal volume was calculated from the segmentation of the SXT 3D reconstruction of the parasite. The total volume of the hemozoin crystals, $V$(Hz), was measured as $2.13^{+0.3}_{-0} \times 10^8$ $nm^3$ considering the missing wedge elongation. The unit cell of a hemozoin crystal has a volume of $v_{UC}$(Hz)=1.407 $nm^3$ (see Straasø et al. [2,4]) and it contains two iron atoms as heme monomers. Thus the iron content in hemozoin crystals was calculated as Fe(Hz)=$2\cdot V(\text{Hz})/v_{UC}(\text{Hz})$.

For the measured $V$(Hz) the number of iron atoms in hemozoin, Fe(Hz), is $3.03^{+0.45}_{-0} \times 10^8$ .

**Estimation of heme crystallization rate in various *in vitro* environments**

Fitch et al.[5] measured heme crystallization in the presence of various lipids known to be present in the parasite (*Plasmodium berghei*). They found the highest rates (38.5 nmol/ml/hr) in the presence of 1-mono-oleoylglycerol. We converted this into heme monomers/sec per average volume of the digestive vacuole ~5 fl as measured from segmented SXT images (parasite #8 + ref[6]), and then multiplied by the measured fraction of hemozoin crystals (60%) that Fitch et al. observed in their reaction to yield the estimate within lipid in Table 2. Fitch et al. found that the rate of hemozoin formation in an aqueous medium was 2% that of lipids, leading to the in vitro rate estimate within aqueous medium in Table 2.

Jani et al. [7] measured 1566 heme monomers/hr formed by HDP, and found an average of 40 zeptomoles HDP per parasite, leading to the *in vitro* rate estimate for HDP in Table 2.

Olafson et al. [8] measured the rate of growth of beta hematin crystals in buffer saturated octanol used to mimic a lipid sub-phase as 0.0008 nm/s. 20 crystals with dimensions 500x50x50 $nm^3$ would grow by 1600 $nm^3$/s, absorbing about 3200 hematin molecules/s.

**X-ray tomography 3D reconstruction**

The collected projection series were aligned using the Bsoft software package [9] and reconstructed using the weighed back-projection algorithm implemented in either the TOMO3D software [10] or the OS-SART algorithm implemented in the TomoJ software package [11] (for analysis of X-ray absorption coefficients in parasite #8).

The three dimensional structure of the infected red blood cells was obtained by manual segmentation of the reconstructed tomograms. The cellular compartments segmented for this study were red blood cell cytosol, parasite cytosol, digestive vacuole, and hemozoin crystals. The segmented compartments were visualized by surface rendering using the Avizo software package.

**Calculation of hemoglobin degradation rate by falcipain 2**

The degradation rate of hemoglobin by falcipain 2 is calculated using the results reported by Chugh et al.[12]

Figure S3(D) in Chugh, et al.[12] shows 75% of hemoglobin digested after 3 hours. 75% of 30 mg of hemoglobin corresponds to $1.97\times10^{14}$ molecules. There are 50nM of falcipain 2 protease concentration in 1 ml (see Methods section in Chugh, et al. [12]) This corresponds to $3\times10^{13}$ falcipain 2 molecules. Hence, the rate of hemoglobin degradation per single falcipain 2 molecule is ~606 hemoglobin molecules per second. There are ~24000 HDP molecules in the malaria parasite according to Jani et al.[7] Assuming there is an equivalent amount of falcipain 2 (since it is complexed with HDP), the overall rate of hemoglobin degradation in the digestive vacuole would be $24000*606=1.45\times10^{7}$ Hb/s

**References**


1 Grimshaw, K., Sahler, J., Spinelli, S. L., Phipps, R. P. & Blumberg, N. New frontiers in transfusion biology: identification and significance of mediators of morbidity and mortality in stored red blood cells. *Transfusion* **51**, 874-880, doi:10.1111/j.1537-2995.2011.03095.x (2011).

2 Straasø, T. *et al.* The Malaria Pigment Hemozoin Comprises at Most Four Different Isomer Units in Two Crystalline Models: Chiral as Based on a Biochemical Hypothesis or Centrosymmetric Made of Enantiomorphous Sectors. *Cryst Growth Des* **14**, 1543-1554 (2011).

3 Hanssen, E. *et al.* Soft X-ray microscopy analysis of cell volume and hemoglobin content in erythrocytes infected with asexual and sexual stages of *P. falciparum*. *J. Struct. Biol.* **177**, 224–232 (2011).

4 Straasø, T. *et al.* The Role of the Four Stereoisomers of the Heme Fe-O Cyclic Dimer in the Crystalline Phase Behavior of Synthetic Hemozoin: Relevance to Native Hemozoin Crystallization. *Cryst. Growth Des.* **11**, 3342-3350 (2011).

5 Fitch, C. D., Cai, G. Z., Shen, Y. F. & Shoemaker, D. J. Involvement of lipids in ferriprotoporphyrin IX polymerization in malaria. *Biochim. Biophys. Acta* **1454**, 31-37 (1999).

6 Kapishnikov, S. *et al.* Oriented nucleation of hemozoin at the digestive vacuole membrane in *Plasmodium falciparum*. *Proc. Natl. Acad. Sci. U. S. A.* **109**, 11188 – 11193 (2012).

7 Jani, D. *et al.* HDP—A Novel Heme Detoxification Protein from the Malaria Parasite. *PLoS Pathog.* **4**, e1000053 (2008).

8 Olafson, K. N., Ketchum, M. A., J.D.Rimer & Vekilov, P. G. Mechanisms of hematin crystallization and inhibition by the antimalarial drug chloroquine. *Proc. Natl Acad. Sci. U.S.A.* **112**, 4946 - 4951 (2015).

9 Heymann, J., Cardone, G., Winkler, D. & Steven, A. Computational resources for cryo-electron tomography in Bsoft. *J. Struct. Biol.* **161**, 232–242 (2008).

10 Agulleiro, J. I. & Fernandez, J. J. Fast tomographic reconstruction on multicore computers. *Bioinformatics* **27**, 582-583 (2010).

11 Messaoudi, C., Boudier, T., Sorzano, C. O. S. & Marco, S. TomoJ: tomography software for three-dimensional reconstruction in transmission electron microscopy. *BMC Bioinformatics* **8**, 288-297 (2007).

12 Chugh, M., Sundararaman, V., Kumar, S., Reddy, V. S. & Siddiqui, W. A. Protein complex directs hemoglobin-to-hemozoin formation in *Plasmodium falciparum*. *Proc. Natl Acad. Sci. U.S.A.* **110**, 5392-5397 (2013).

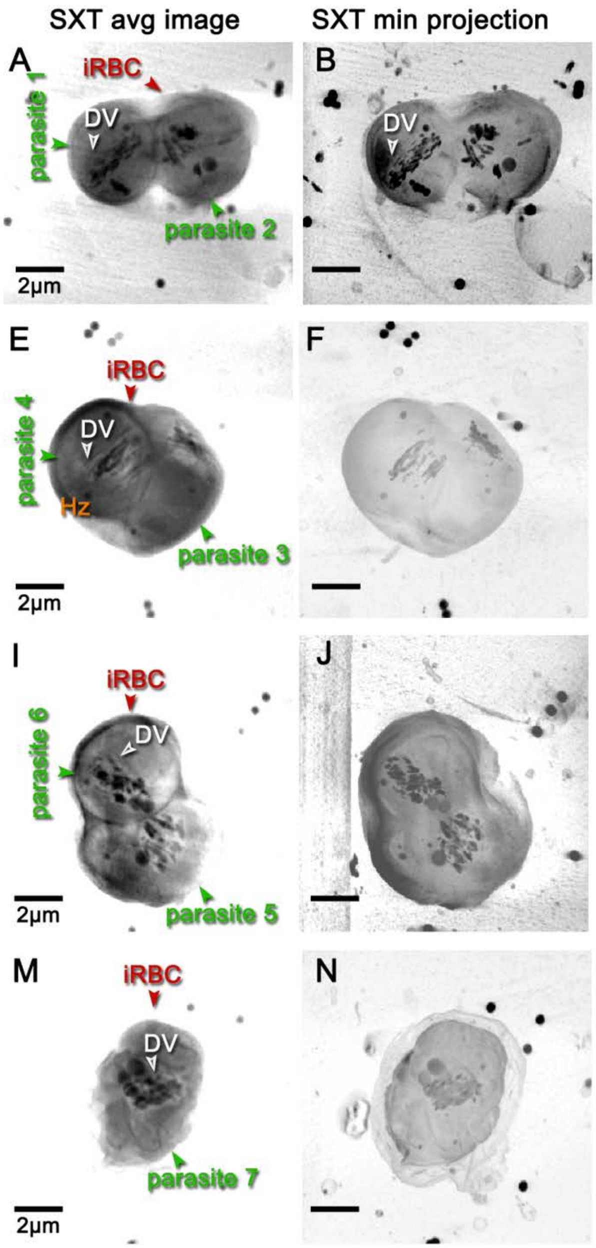


**Supplementary Figure 1** Increased contrast in SXT projections shown in Fig. 2 in the main text to help visualize the parasite digestive vacuole

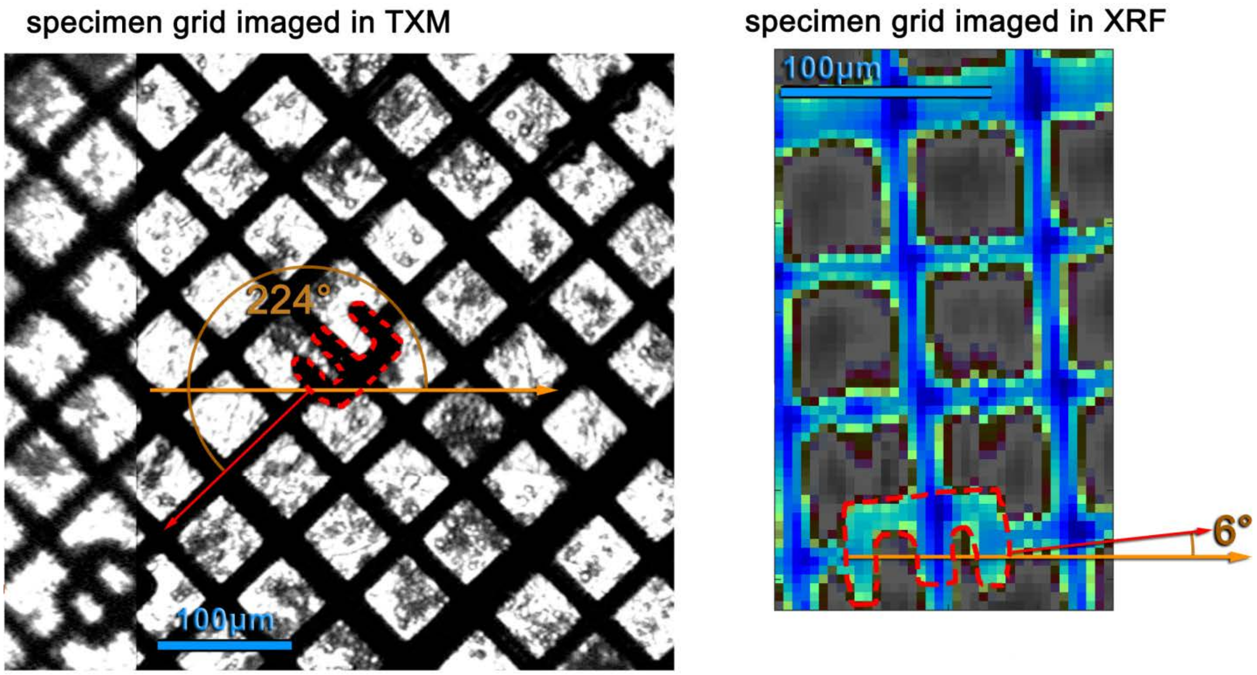


**Supplementary Figure 2** Orientation of the specimen grid in the transmission X-ray microscope (TXM), left, and in the X-ray fluorescence microscope, right. Letter “E” printed on the specimen grid is highlighted with a dotted red line. The in-plane orientation of the grid for both cases is shown in degrees.

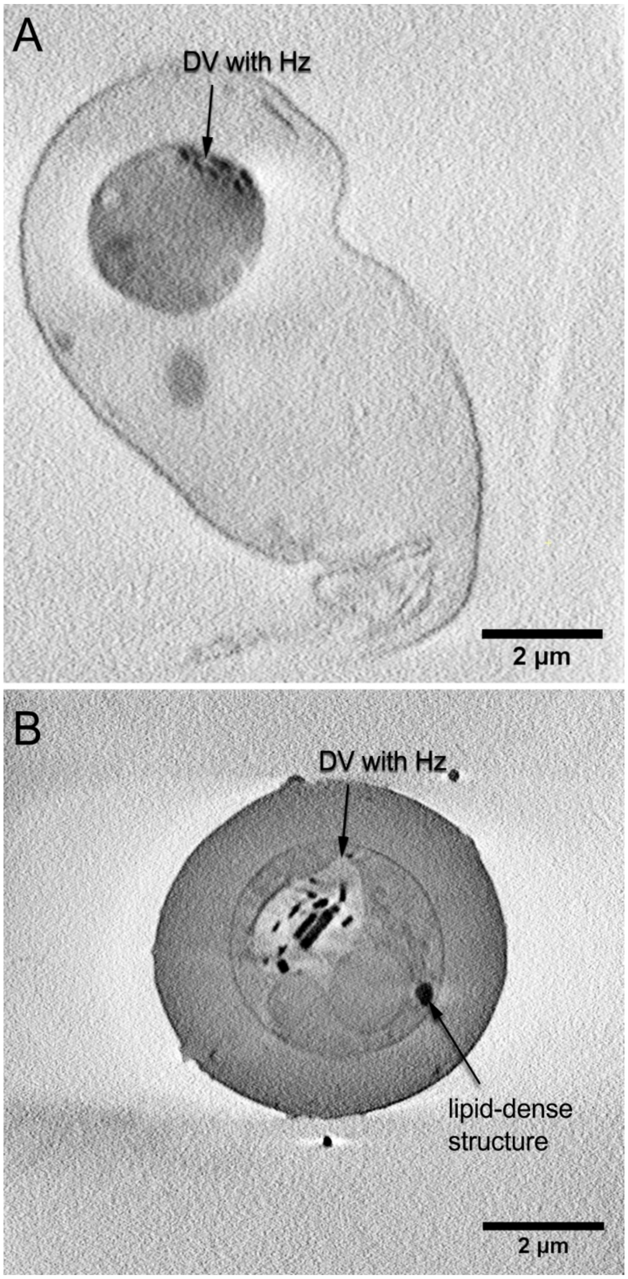


**Supplementary Figure 3** Soft X-ray tomography of *P. falciparum* infected red blood cells. (A) A 13 nm thick virtual slice through a trophozoite featuring digestive vacuole with small hemozoin crystals. Imaged using a 41 nm zone plate. (B) An 11.3 nm virtual slice through a schizont within an infected red blood cell. Two nuclei, a lipid-dense structure as well as the digestive vacuole with hemozoin crystals are clearly visible in this slice. Imaged using a 25 nm zone plate.
